# Perinatal Hyperoxia and Developmental Consequences on the Lung-Brain Axis

**DOI:** 10.1155/2022/5784146

**Published:** 2022-02-24

**Authors:** Stefanie Obst, Josephine Herz, Miguel A. Alejandre Alcazar, Stefanie Endesfelder, Marius A. Möbius, Mario Rüdiger, Ursula Felderhoff-Müser, Ivo Bendix

**Affiliations:** ^1^Department of Paediatrics I, Neonatology and Experimental Perinatal Neurosciences, University Hospital Essen, University Duisburg-Essen, 45147 Essen, Germany; ^2^Centre for Translational Neuro- and Behavioural Sciences, C-TNBS, Faculty of Medicine, University Duisburg-Essen, 45147 Essen, Germany; ^3^Cologne Excellence Cluster for Stress Responses in Ageing-Associated Diseases (CECAD) and Center for Molecular Medicine Cologne (CMMC), University of Cologne, Faculty of Medicine, University of Cologne, 50931 Cologne, Germany; ^4^Institute for Lung Health, Member of the German Centre for Lung Research, University of Giessen and Marburg Lung Center, 35392 Giessen, Germany; ^5^Department of Pediatric and Adolescent Medicine, University of Cologne, Faculty of Medicine, University of Cologne, 50937 Cologne, Germany; ^6^Department of Neonatology, Charité-Universitätsmedizin Berlin, 13353 Berlin, Germany; ^7^Department for Neonatology and Pediatric Intensive Care, Clinic for Pediatric and Adolescence Medicine, Faculty of Medicine, Technische Universität Dresden, 01307 Dresden, Germany; ^8^Saxony Center for Feto-Neonatal Health, Faculty of Medicine, Technische Universität Dresden, 01307 Dresden, Germany

## Abstract

Approximately 11.1% of all newborns worldwide are born preterm. Improved neonatal intensive care significantly increased survival rates over the last decades but failed to reduce the risk for the development of chronic lung disease (i.e., bronchopulmonary dysplasia (BPD)) and impaired neurodevelopment (i.e., encephalopathy of prematurity (EoP)), two major long-term sequelae of prematurity. Premature infants are exposed to relative hyperoxia, when compared to physiological in-utero conditions and, if needed to additional therapeutic oxygen supplementation. Both are associated with an increased risk for impaired organ development. Since the detrimental effects of hyperoxia on the immature retina are known for many years, lung and brain have come into focus in the last decade. Hyperoxia-induced excessive production of reactive oxygen species leading to oxidative stress and inflammation contribute to pulmonary growth restriction and abnormal neurodevelopment, including myelination deficits. Despite a large body of studies, which unraveled important pathophysiological mechanisms for both organs at risk, the majority focused exclusively either on lung or on brain injury. However, considering that preterm infants suffering from BPD are at higher risk for poor neurodevelopmental outcome, an interaction between both organs seems plausible. This review summarizes recent findings regarding mechanisms of hyperoxia-induced neonatal lung and brain injury. We will discuss common pathophysiological pathways, which potentially link both injured organ systems. Furthermore, promises and needs of currently suggested therapies, including pharmacological and regenerative cell-based treatments for BPD and EoP, will be emphasized. Limited therapeutic approaches highlight the urgent need for a better understanding of the mechanisms underlying detrimental effects of hyperoxia on the lung-brain axis in order to pave the way for the development of novel multimodal therapies, ideally targeting both severe preterm birth-associated complications.

## 1. Introduction

Premature birth < 37 weeks of gestation affects approximately 11.1% of all newborn infants worldwide and is one of the leading causes of infant mortality and long-term morbidity [1, 2]. Over the last decades, significant advances in perinatal, obstetric, and neonatal care increased survival of very premature born infants, e.g., <29 weeks of gestation [3]. However, improved survival was not associated with a reduction of long-term sequelae including encephalopathy of prematurity (EoP), retinopathy of prematurity (RoP), and chronic lung diseases such as bronchopulmonary dysplasia (BPD), all associated with life-long individual, familial, financial, and socioeconomic burden [4–7]. With preterm birth, the extra-uterine fetus is exposed to relative hyperoxia with a partial oxygen tension of approx. 70 mmHg compared to 25 mmHg in utero. Additionally, the most common treatment for premature infants with an immature lung is the use of supplemental oxygen and/or mechanical ventilation, further exacerbating the hyperoxic effect [8, 9]. Though improved guidelines and associated changes in clinical routine led to a reduction of invasive ventilation, the frequency of chronic lung disease remained high [10].

Elevated oxygen exposure is associated with developmental disturbances of both, the immature lung and brain, characterized by simplification of alveolar and vascular growth in the lung and white matter injury in the brain, major characteristics of BPD and EoP, respectively [11–13]. Although oxidative stress and inflammatory reactions following neonatal hyperoxia were identified as key contributors to the pathogenesis of BPD and EoP, the cellular and molecular mechanisms are not fully understood [14, 15]. BPD is a multifactorial chronic disease associated with several comorbidities like cardiovascular diseases and RoP [16, 17]. Furthermore, BPD seems to be a predictor of poor neurodevelopmental outcome following EoP. However, possible pathways linking prematurity associated brain and lung damage are only poorly understood [18–20]. It still remains unclear whether EoP and BPD are symptoms of a common pathway induced by a unique injury or whether brain injury develops secondary to primary injury of the lung via lung-brain axis.

Even though experimental and clinical studies suggested several pharmacological interventions for the separate treatment of BPD and EoP, there are no common therapies for prevention or treatment of both interrelated preterm birth associated complications in standard care [21, 22]. Recently, regenerative stem cell-based therapies gained much interest as therapeutic approaches to treat immature lung and brain injury. However, their potential to improve both organ injuries with the same treatment regime remains unclear [23, 24]. In this review, we provide an overview about current knowledge of pathophysiological mechanisms of lung and brain injury, associated with prematurity. Further, we will discuss differences, similarities, and the gap of knowledge regarding cellular and molecular pathways. Therapeutic approaches, potentially applicable to treat both, neonatal lung, and brain injury, will be outlined.

## 2. Experimental Models of BPD and EoP

**Figure 1:**
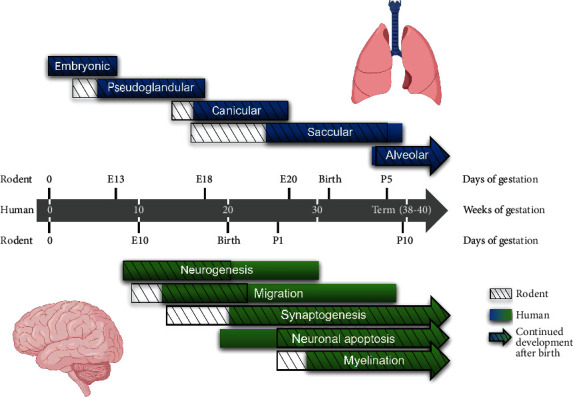
Comparison of lung and brain development in humans and rodents. The time course of lung (upper panel, blue) and brain (lower panel, green) development of humans (filled bars) and rodents (shaded overlays) is shown during gestation and the neonatal period (bars with arrowheads indicate continued development after birth). This summary was created based on previous reports for lung [27, 36] and brain [35, 184] development.

The multifactorial origins of BPD and EoP have been intensively investigated in a large number of animal models including rodents [25–27], rabbits [28, 29], baboons [30, 31], and sheep [32, 33]. In rodent models, the developmental stage of the lung and the brain in newborn pups is comparable with preterm humans (Figure 1). The period of rapid brain growth in humans takes place in the last trimester of pregnancy proceeding until two years of age whereas the growth spurt in neonatal rodents is delayed to postnatal day 2 (P2) to P10 [34, 35]. Similarly, human premature infants are usually born in the saccular phase of lung development, which takes place in rodents from E17/E18 to P4/P5 [27, 36] (Figure 1). The impact of hyperoxia on brain and lung injury was analyzed in several preclinical and clinical studies [22, 37–44], but only very few preclinical studies demonstrated effects on both organs in the same experimental model [45–48]. This may be partially explained by huge variations in hyperoxia onset, duration, and oxygen concentrations between experimental models (Table 1). In most models of EoP, an oxygen concentration of 80% is used whereas >85% oxygen is used in the majority of BPD studies. Even more striking is the difference in the duration of hyperoxia with mainly 10 to 14 days for lung injury compared to a short period of 6 h to 48 h in brain injury models (Table 1). Furthermore, onset of short-term hyperoxia to assess brain injury was between P3 and P6 [49–57], while the majority of studies analyzing oxygen-induced lung injury exposed newborn rodents from the day of birth onwards [58–67]. Nevertheless, some studies also examined the brain after longer oxygen exposure [44–48, 68–71], demonstrating an impaired white matter development due to increased inflammatory reactions and cell death of developing oligodendrocytes, similarly as observed in short-term hyperoxia-studies [55–57, 72–75]. Notably, hyperoxia was started at timepoints around birth to P2 in these long-term hyperoxia studies, which closely corresponds to experimental BPD studies (Table 1) [27, 48]. Therefore, recent rodent models of EoP and BPD may be combined for simultaneous analyses of hyperoxia-induced lung and brain injury to entangle potential interrelated pathways linking both organ pathologies and to screen for possible common therapeutic approaches. Nevertheless, several factors have to be taken into account when interpreting data and comparing studies. For example, experimental models demonstrated that the severity and phenotype of lung injury depend on the concentration and duration of oxygen exposure [76]. Furthermore, characteristic pathological features and molecular mechanisms vary between mouse strains. Therefore, strain-dependent effects and genetic susceptibility need to be considered for reproducibility and reliability of results in experimental models of hyperoxia-induced injury [77, 78].

Despite of the advantages of rodent models (e.g., short life span enabling large sample sizes within a short time period, inbred strains with less variability, and large litter sizes), the clinical relevance of the results has to be interpreted with caution. Whereas developmental stage of the lung and the brain in newborn rodents is comparable with preterm humans, alveolarisation and brain development take place ex-utero, e.g., under room-air conditions with 21% of oxygen. In contrast, in humans, these processes are programmed to take place in utero, i.e., under “hypoxic” conditions. Thus, even at room-air (21% oxygen), the human preterm should be considered as an extra-uterine fetus that is exposed to relative hyperoxia with a deleterious effect. This very specific situation is better simulated in large animal models of prematurity, which are of utmost importance for clinical translation. These models, inducing preterm birth either by hysterotomy or caesarean section, enable assessment of the impact of invasive ventilation and other hits like chorioamnionitis, maternal, or fetal inflammation, all of them supposed to contribute to detrimental development of the immature lung and brain [79–81]. Furthermore, physiological parameters like oxygen saturation, blood pressure, exhaled gas levels (e.g., NO), tidal volume, airway pressure, and blood gas analysis [82, 83] are easier to analyze in large animal models. In order to increase our understanding of the effect of prematurity on fetal organ development and to improve translation of experimental findings to neonatal intensive care, data from large animal models like nonhuman primates, piglets, or lambs are urgently needed [30, 84, 85].

## 3. Hyperoxia-Induced Oxidative Stress in the Immature Lung and Brain

**Figure 2:**
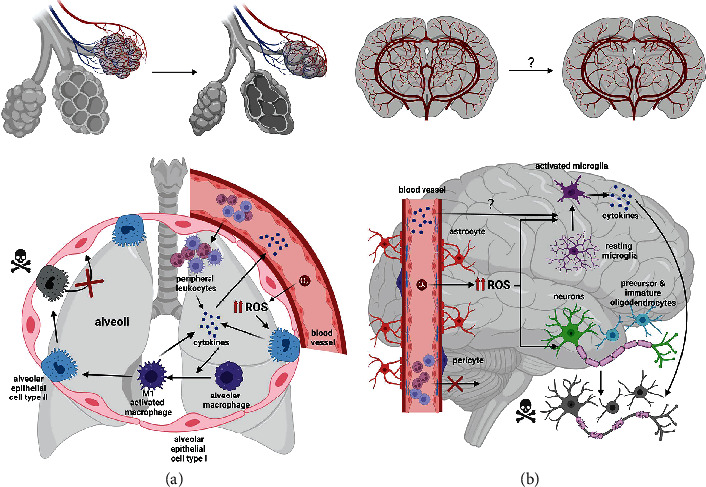
Hyperoxia-induced morphological changes and inflammatory responses in the developing brain and lung. Hyperoxia disrupts alveolar and vascular development in the immature lung resulting in fewer and larger alveoli and decreased vessel density ((a) upper panel). With regard to mechanisms underlying impaired lung development, enhanced ROS production stimulates alveolar epithelial cells type II (AECII) to produce proinflammatory cytokines (IL6, IL-18, IL-1beta, TNF-alpha, etc.) resulting in infiltration of peripheral leukocytes (macrophages, neutrophils, monocytes, etc.) ((a) lower panel). Detrimental effects of proinflammatory cytokines were ascribed to activation and polarization of alveolar and peripheral macrophages into proinflammatory M1 macrophages, which not only accelerate proinflammatory cytokine production but also lead to degeneration of AECII cells and reduced developmental transition from AECII into AECI. These mechanisms may contribute to reduced formation of alveoli. In the developing brain, first evidences suggest that hyperoxia impairs vascularization, though this needs to be proven in future studies ((b) upper panel). Similarly to the lung, hyperoxia leads to increased oxidative stress through enhanced ROS production ((b) lower panel). Increased ROS have detrimental effects on oligodendrocyte maturation, myelination, and neuronal survival, leading to ultrastructural abnormalities of myelin formation and grey matter injury ((b) lower panel). Furthermore, increased ROS in the brain activate microglia cells, associated with proinflammatory cytokine expression (IL-18, IL-1beta, TNF-alpha, etc.), thereby additionally enhancing both white and grey matter injury. In contrast to hyperoxia-injured lungs, peripheral leukocytes do not infiltrate the brain, most likely due to protection by unique characteristics of the blood-brain barrier.

Under physiological conditions, the excess of reactive oxygen species (ROS) leading to oxidative stress is counterbalanced by a tightly regulated system of antioxidative enzymes and radical scavengers. Oxidative stress is one major factor contributing to hyperoxia-induced injury in both, the developing lung and brain (Figure 2). Preterm infants are very susceptible to oxidative stress due to their immature antioxidant defense, leading to increased production of ROS like superoxide (O_2_^•^), hydrogen radicals (HO^•^), and hydrogen peroxide (H_2_O_2_). Under hyperoxic conditions, increased ROS triggers DNA damage, protein, and lipid oxidation resulting in altered physiological properties and function of specific cell types and developing organs [14, 86]. Antioxidant enzymes preserving the cell against oxidative damage include glutathione peroxidase (GPx), catalase, and superoxide dismutase (SOD). SODs, localized in the cytoplasm (SOD1), mitochondria (SOD2) or secreted into the extracellular space (SOD3), convert O_2_^•^ into H_2_O_2_, which is less reactive than the free oxygen radical [87]. In neonatal rats, hyperoxia induces a decrease of SOD1 and 3 in the brain [51]. Similarly, in hyperoxia-injured lungs, SOD1-3 expression is significantly reduced after 3 and 5 days of hyperoxia, which persists until 10 days of recovery under normoxic conditions [59]. These studies indicate similar oxidative stress responses in the developing brain and lung. In support of this, lung-specific overexpression of SOD3 did not only protect against the arrest of alveolar proliferation but also improved short-term memory in female adult mice exposed to neonatal hyperoxia, supporting the hypothesis of an inter-organ communication [58, 68]. With regard to the underlying molecular mechanisms, SOD1 and 2 expression is regulated by the redox-sensitive transcription factor Nrf2 (nuclear factor erythroid 2-related factor 2), a key regulator of antioxidative and inflammatory responses and a mediator of important cellular processes like maturation and proliferation [88]. Under physiological (normoxic) conditions, Nrf2 is inhibited by Kelch-like ECH-associated protein-1 (Keap1). However, in case of oxidative stress, Nrf2 is released from its inhibitor and induces antioxidant gene expression including SOD1 and 2, hemeoxygenase-1 (HO-1), and GPx [89]. Recent work in lung and brain injury showed that hyperoxia increases Nrf2 in both lung and brain, whereas downregulation of the counterpart Keap1 is only detected in lung tissues [51, 59]. These data support previous work revealing that Nrf2 activity can be regulated in a Keap1-dependent and –independent manner under oxidative stress [88]. Differences in Keap1 regulation also indicate a lung-intrinsic antioxidant mechanism with increased availability of Nrf2 after hyperoxia. Furthermore, different levels of oxidative stress in lung and brain tissues, organ-specific regulatory antioxidant mechanisms, or time-dependent different dynamics in response to hyperoxia may account for the observed differences, suggesting a possible lung to brain sequence of injury. Nevertheless, it should be taken into account that the onset and duration of oxygen exposure also differed in the aforementioned studies (i.e., lung P0 to P3/P5 vs. brain P6 to P7) [51, 59].

Even though H_2_O_2_ is less reactive than free oxygen radicals, it is toxic to lung and brain cells. The oxidative effect of H_2_O_2_ is attenuated through reduction of H_2_O_2_ to O_2_ and H_2_O by GPx, which oxidizes glutathione (GSH) to glutathione disulfide (GSSH). GSH is recovered by glutathione reductase [86]. As a measure of hyperoxia-induced oxidative stress in perinatal brain injury, we reported decreased GSH levels and elevated GSSH levels [43, 57], which were associated with increased acetylcholinesterase expression implicating stress-induced alterations of cholinergic neurotransmission by these biochemical pathways [73]. Disturbances in GSH/GSSH levels were associated with oligodendrocyte maturation and long-term myelination deficits, thereby most likely contributing to impaired long-term neurodevelopmental outcome [43, 55, 75, 90]. Similar results were obtained in lung tissues, shown by a significant increase of GSSH [91, 92], though a correlation to lung-function was not evaluated in these studies.

In addition to antioxidative enzymes discussed above, other important oxygen scavenger enzymes are nitric oxide synthases, which produce nitric oxide (NO). NO is a multifactorial signaling molecule that influences many physiological and pathological processes including immunoregulation, neuronal transmission, platelet aggregation, airway branching, and pulmonary vascularization [93–95]. However, endogenous NO, produced by nitric oxide synthases (NOS), also reacts with oxygen radicals, which subsequently enhances nitric stress due to production of reactive nitric species [14]. Three isozymes of NOS have been described: inducible NOS (iNOS), neuronal NOS (nNOS), both are soluble and primarily located in cytoplasm, and endothelial NOS (eNOS), which is membrane associated. eNOS and nNOS are constitutively expressed and calcium-dependent, while iNOS is calcium-independent and only induced under certain proinflammatory conditions [96, 97]. Interestingly, hyperoxia exposure triggers an increased expression of iNOS in endothelial and perivascular cells in the cortex and in microglial cells in the hippocampus in neonatal rats [98]. Furthermore, Sirinyan and colleagues noticed eNOS and nNOS upregulation in cerebral capillaries, which in combination with reduced SOD1 expression may contribute to cerebral microvasculature injury in the developing brain [44]. In contrast, a significant reduction of all NOS isoforms was reported in lung tissues of ventilated premature baboons [82]. Furthermore, eNOS was decreased in neonatal rat and mouse lungs, indicating a different nitric stress response to hyperoxia in lung and brain tissues [64, 99]. These data emphasize the need to delineate similarities and differences between both organ pathologies in one and the same experimental model system to improve our understanding of basic mechanisms.

## 4. Inflammatory Responses in Hyperoxia-Induced Neonatal Lung and Brain Injury

Both organ injuries share significant similarities regarding inflammatory mechanisms, which is reflected by upregulation of a variety of proinflammatory cytokines, like TNF-alpha, IL-1beta, IL-6, and IL-18 [14, 51, 53, 60, 73, 100, 101]. As underlying mechanisms, ROS-mediated activation of transcription factors, such as nuclear factor kappa-light-chain-enhancer of activated B-cells (NF-kappaB), a key mediator in stimulation of inflammatory responses, is supposed to induce proinflammatory cytokine expression in both organs [51, 59, 102]. An additional mechanism might be that proinflammatory cytokines produced in the lungs enter the circulation and cross the blood-brain barrier (BBB) to accelerate hyperoxia-induced inflammatory responses. This is supported by our previous work, demonstrating that the lung is a large source of systemic TNF-alpha production [103, 104]. A further cytokine-related mechanism linking both organ systems might be IL-6 signaling. Pharmacological inhibition of global IL-6 signaling and IL-6 trans-signaling improved survival and alveolarization in hyperoxia-induced lung injury [60]. It remains to be elucidated whether this approach may have equal beneficial effects in the brain. Nevertheless, it should also be taken into account that a certain level of inflammation might be needed for physiological organ development, as indicated at the example of disruption of NF-kappaB signaling, which was shown to contribute to the pathogenesis of BPD [105], highlighting that a balanced inflammatory signaling is important for alveolarization.

In the neonatal brain, hyperoxia-induced cell death was associated with an increased mRNA and protein expression of caspase-1, IL-1beta, and IL-18, all of them involved in inflammasome-associated signaling [53]. Of note, inhibition of IL-18 with an intraperitoneal administration of human IL-18-binding protein diminished hyperoxia-induced brain injury [53]. Furthermore, inhibition of caspase-1, a major component of the NOD-like receptor domain-containing protein 1 (NLRP1) inflammasome, attenuated hyperoxia-induced NLRP1 inflammasome activation, which was associated with reduced cerebral atrophy and cell death as well as increased proliferation in the neurogenic subventricular and subgranular zone [47]. With regard to lung injury, induced by hyperoxia, inflammasome signaling seems to play a similarly important role. Caspase-1 inhibition leads to a decreased production of mature IL-1beta, which was associated with reduced infiltration of macrophages, improved alveolarization and vascular architecture, and a reduced right ventricular hypertrophy [47]. In addition to NLRP1, hyperoxia leads to activation of NLRP3 in both organs [106, 107]. While this inflammasome was shown to play a crucial role in the development of BPD [106], its functional relevance in hyperoxia-induced brain injury remains to be investigated.

Hyperoxia-induced inflammatory responses include activation and modulation of resident immune cells in both organs, i.e., microglia and alveolar macrophages (Figure 2). In the developing brain, hyperoxia leads to increased microglia activity, demonstrated by increased ionized calcium-binding adapter molecule 1 (Iba-1) protein expression, which was associated with increased IL-1beta release [57, 108]. Inhibition of these early inflammatory responses by minocycline administration revealed protective effects on proliferation of oligodendroglia precursor cells [108]. Concerning the lung, recent studies by Domingo-Gonzalez and colleagues highlighted the diversity of the lung immune system and identified a variety of specialized immune cells, including dynamic regulation of macrophage subtypes [109]. For instance, hyperoxia stimulates the transdifferentiation of resident alveolar macrophages into activated macrophages (CD45^+^ CD11c^+^ SiglecF^+^ CD11b^+^ CD68^+^ MHCII^+^), which may contribute to impaired alveolar growth in neonatal lungs [61]. Another recent study showed hyperoxia-induced activation of proinflammatory M1 lung-macrophages, which was related to a reduction of the transcription factor Krüppel like factor 4 (Klf4) *in vitro* and a decreased survival of type II alveolar epithelial cells (AECII) *in vivo* [60]. This may lead to impaired lung development, since AECII play a crucial role in alveolar recovery after lung injury due to their high self-renewal properties [110].

In addition to resident immune cells, peripheral immune cells are involved in BPD pathogenesis. Hyperoxia-injured lungs showed a dramatic infiltration of peripheral leukocytes including neutrophils, monocytes, and macrophages, resulting in harmful effects on endothelial and epithelial cells and increased ROS production, all of them supposed to contribute to cell death and arrest of lung growth (Figure 2(a)) [91, 100, 111]. In contrast to the lung, hyperoxia-induced brain injury was not associated with an infiltration of peripheral immune cells (Figure 2(b)) [57]. This is possibly due to the unique properties of the blood-brain barrier (BBB), which protects the brain against nonselective transmission of solutes and cells. Despite the widespread assumption of an immature and leaky BBB in preterm and term infants, immunohistochemical analyses in E16 rat brains suggested a functional and fully intact BBB [112]. Nevertheless, severe perinatal insults in the developing brain such as hypoxia ischemia, focal arterial stroke, or inflammation impair BBB integrity resulting in higher traffic of small proteins, which may disturb neurodevelopment [113]. Effects of hyperoxia on neurovascular unit development still remain unclear and need to be investigated in future studies.

## 5. Effect of Hyperoxia on Vascular Formation and Structural Remodeling in the Developing Lung and Brain

Oxidative stress and inflammation triggered by hyperoxia have a detrimental effect on morphology of the developing lung and brain. Lung vascular development and alveolar growth are highly interrelated. BPD leads to a decrease of lung microvasculature architecture, which persists into adulthood [63, 114]. Reduced vessel densities, abnormal vessel distribution, reduction of small arteries, and alveolar simplification (Figure 2(a)) are frequently observed in infants suffering from BPD and in experimental models [63, 115–117]. Different underlying signaling pathways have been suggested to induce abnormal lung growth [118]. For instance, transforming growth factor-beta (TGF-beta) signaling, which is crucial in vascular and associated lung development [62, 119], is altered under hyperoxic conditions. S-endoglin, the short isoform of endoglin (TGF-beta type III receptor), which regulates phosphorylation of TGF-beta type II and type I receptors, is upregulated by hyperoxia, leading to stimulation of TGF-beta activin-like kinase 5-SMAD2/3 signaling, finally resulting in alveolar simplification and decreased vessel density in the developing lung [62]. In support of this, blocking TGF-beta signaling protected from hyperoxia-induced injury and enabled lung growth [120]. Hyperoxia-induced vascular abnormalities noticed in experimental models and affected BPD-infants is also accompanied by a reduction of proangiogenic factors like vascular endothelial growth factor (VEGF), its receptors VEGFR1 and VEGFR2 as well as angiopoetin-1 receptor (Tie2) [64, 114]. Inhibition of VEGF leads to BPD-like impaired alveolar and vascular architecture in neonatal rats, while gene therapy with a recombinant adenovirus, carrying the VEGF-gene (gene treatment), improved lung development [64]. Interestingly, the protective effect of VEGF can be enhanced by a combination of VEGF and angiogenin-1 gene treatment, resulting in an improved vessel architecture with less newly formed fenestrated vessels compared to VEGF gene treatment alone [64]. VEGF is a major target gene of hypoxia-inducible factor (HIF), which plays an important role in fetal organ development. By exposing the fetus to an extra-uterine environment, the associated relative hyperoxia will cause a destabilization of HIF, which has antiangiogenic effects. This was recently demonstrated by Vadivel et al., who showed that HIF-inhibition does not only downregulate the expression of HIF1-alpha and HIF2-alpha but also of VEGF, which was associated with reduced vessel density and less alveolarization in the developing lung [65]. The importance of HIF signaling in vascularization is further shown in a study inhibiting HIF-activation by prolyl-hydroxylases, which led to enhanced levels of proangiogenic factors like VEGF and platelet endothelial cell adhesion molecule and was associated with improved angiogenesis [121]. In addition to VEGF, the proangiogenic factor apelin was shown to promote alveolarization and to protect from hyperoxia-induced lung injury [122].

While impaired vascularization in hyperoxia-induced BPD models is well described, far less is known about the impact of hyperoxia on vascular development in the brain. Sirinyan et al. showed a reduction of cortical microvessels in neonatal rats after 6 days of hyperoxia, which persisted into adolescence [44]. Diminished vessel density was associated with an early increase of nitric stress, which may have contributed to a downregulated expression of VEGFR2 and impaired neurocognitive functional outcome [44]. Interestingly, Morken and colleagues found an increased cortical vascular network in rat pups in a model of neonatal intermitted hyperoxia-hypoxia and speculated that hyperoxia-induced vasoobliteration triggers hypoxic angiogenesis [123]. Furthermore, vascular abnormalities were associated with altered white matter development, demonstrated by higher mean, axial and radial diffusivity, and a lower fractional anisotropy (FA) at P14 in diffusion tensor imaging. [123]. Of note, these alterations in white matter development were not observed at P28, indicating a maturation delay of the white matter. Similar effects were observed in experimental models of pure hyperoxia for 24 hours, though vascularization was not analyzed in these studies [49, 55, 57, 90]. Further research is needed to decipher the exact relationship between developmental vascularization and myelination. Furthermore, due to first evidences, that severe BPD is associated with poor neurodevelopmental outcome [18], further investigations are needed to analyze whether and how hyperoxia-induced structural abnormality in the lung is causally linked to brain injury, i.e., neurodevelopmental outcome [124].

In addition to alterations of vascularization, hyperoxia causes pronounced structural deficits leading to organ dysfunction in the developing lung and brain. Impaired vascularization in the lung is associated with lung growth restriction including arrest of alveolarization. In addition to an overall reduced number of alveoli and alveolar surface, larger mean linear intercept (mean distance between two alveolar septae) and reduced radial alveolar count (fewer but larger alveoli) have been frequently reported [76, 125], even though the latter two parameters are still debated. Therefore, it is of outstanding importance to analyze these heterogeneous structural alterations in the lungs using unbiased methods, for example, design-based stereology or whole-organ imaging approaches [126, 127]. Further structural changes include an increased septal thickness, enhanced collagen and smooth muscle actin content, and diffuse distribution of elastin, all together impairing respiratory volume and function [11, 60, 119, 128, 129]. Although the exact mechanisms underlying lung growth arrest after hyperoxia are still not entirely clear, different cell types were extensively studied. For instance, different fibroblast subpopulations were identified contributing to alveolar formation, injury, and repair. Notable amongst those are lipofibroblasts, a specialized fibroblast subtype that aligns with AECII and support their homeostasis and differentiation [130–132]. Even though hyperoxia stimulates an acute increase of AECII, this is followed by depletion of these cells under normoxic recovery conditions, which lasts into adulthood. The secondary AECII loss may contribute to reduced formation of alveoli, though the cause of AECII loss during recovery is still unknown and needs further investigation [67]. Further open questions include the impact of TGF-beta signaling in fibroblasts on modulation of the extracellular matrix and subsequent alveolar growth arrest in hyperoxic lungs [119]. Finally, the role of other lung cells, e.g., alveolar epithelial cells type I in hyperoxia-induced lung injury remains elusive.

In comparison to structural remodeling of the entire immature lung, hyperoxia affects specific brain regions, i.e., hippocampus, cortex, white matter, striatum, caudate nucleus, and cerebellum [43, 52, 53, 133–136]. The most prominent feature of hyperoxia-induced brain injury is cell death of precursor and immature oligodendrocytes associated with a reduction of mature oligodendrocytes [49, 57, 74, 75, 90, 134, 135, 137]. This leads to a decreased expression of myelin-related proteins like myelin basic protein (MBP) resulting in subacute hypomyelination and long-term ultrastructural myelin abnormalities, which were associated with neurocognitive deficits [49, 55, 57, 90]. In addition to myelination deficits, hyperoxia led to increased neuronal cell loss and impaired hippocampal neurogenesis. [43, 52, 53, 133, 136]. Therefore, both, white and grey matter injury might contribute to hyperoxia-induced neurodegeneration [43, 53, 90], though cellular causes and consequences remain to be delineated.

With regard to the lung-brain axis, Kim and colleagues showed that hypoalveolarization was associated with hypomyelination and increased neuronal cell death [45]. Furthermore, increased proinflammatory cytokines and decreased VEGF expression in lung tissues correlated with reduced brain weight, supporting the hypothesis that lung injury may aggravate adverse neurodevelopmental outcome [45]. A recent study revealed that extracellular vesicles/exosomes released from AECII cells in hyperoxia-injured lungs induced lung and brain injury when adoptively transferred into naïve neonatal rats [46]. The authors suggested that these extracellular vesicles/exosomes may enter the circulation, subsequently cross the BBB, and induce inflammatory brain injury [46]. These studies support the hypothesis of a detrimental interaction between lung and brain injury caused by hyperoxia.

## 6. Potential Pharmaceutical Approaches for Treatment of Neonatal BPD and EoP

Currently, there is no therapy for prevention and recovery from hyperoxia-induced BPD and EoP. A large number of preclinical studies analyzed the potential of pharmaceutical interventions like caffeine, inhaled NO (iNO), and erythropoietin (Epo) [21, 48, 138–142]. Caffeine, currently one of the most frequently used pharmaceutical treatment in neonatal care, is a nonspecific adenosine receptor antagonist with anti-inflammatory and antiapoptotic properties in brain and lung injury caused by hyperoxia [143]. In hyperoxia-induced neonatal brain injury, caffeine reduces oxidative stress associated with enhanced SOD1-3 mRNA and decreased iNOS expression. These antioxidative effects were related to diminished pro-inflammatory cytokine expression and reduced expression of apoptotic molecules, like apoptosis-inducing factor, cleaved caspase-3, and poly (ADP-ribose) polymerase-1 [51]. Similarly, to its neuroprotective effects, caffeine showed protective effects in hyperoxia-induced lung injury, revealed by reduced oxidative DNA damage and anti-inflammatory effects, e.g., downregulation of chemokine and inflammatory cytokine expression and a decreased pulmonary recruitment of neutrophils and macrophages [59, 100, 102]. Even though the promising agent caffeine has been proven to reduce rates of BPD and intraventricular hemorrhage, the optimal treatment design regarding dose and timing is still under debate in the clinical setting [143].

NO plays a crucial role in pulmonary vascularization, airway branching, and neuronal transmission [93–95]. Due to its neuroprotective and proangiogenic properties [144, 145], it has been suggested that inhaled NO (iNO) may have therapeutic properties. This is supported by experimental studies showing that iNO applied directly after hyperoxia ameliorated disrupted structural development of the lung demonstrated by an increased radial alveolar count [146]. However, Pham and colleagues showed only a transient protective effect, not persisting until the age of P10 [48]. Both studies used a similar low dose of iNO, but different timing of administration (early during hyperoxia [48] vs. late after hyperoxia [146]), which may explain the different outcomes on lung injury. Nevertheless, simultaneous analysis of brain injury in the setting of Pham et al. revealed a neuroprotective effect of iNO, including enhanced density of mature oligodendrocytes and myelination in the developing white matter associated with improved learning scores [48]. Therefore, iNO might represent a common therapeutic strategy to target both, neonatal lung and brain injury. Since iNO is supposed to act locally in the lung instead of being directly transported into the brain, systemic effects of iNO through the circulation affecting the lung-brain axis seem a plausible explanation for protective effects in both organs. Nevertheless, it is intriguing that additionally added NO does not aggravate oxidative stress in the developing lung and brain, which would be expected in hyperoxic conditions. This puzzling aspect is also supported by a cochrane review of 17 randomized clinical controlled trials of iNO therapies in premature born infants showing no or only poor improvements of mortality, survival (without BPD), brain injury, and neurological outcomes [147]. Therefore, further experimental and clinical studies are required analyzing dose, timing, and subgroups of infants/animals with different disease severity states to clearly identify the protective potential for each pathology [147].

In addition to caffeine and iNO, erythropoietin (Epo) also has antiapoptotic and proangiogenic properties [148, 149]. Endogenous Epo is produced by many cell types like neurons, oligodendrocytes, microglia, and astrocytes and its receptors are widely expressed in lung and brain tissues, indicating potential targets for protection of hyperoxia-mediated tissue damage in both organs [150, 151]. Single and repetitive Epo treatment in short-term hyperoxia models showed improved long-term memory deficits in adolescent and adult rats associated with attenuation of hypomyelination and improved neurocognitive outcome [50, 152]. Similarly, in hyperoxia-induced lung injury, low-dose (400 U/kg) Epo therapy resulted in improvements of alveolarization and a higher microvessel count [148]. However, high dose (5000 IU/kg) Epo exacerbated short-term lung injury in ventilated premature lambs caused by increased systemic inflammation, higher airway wall thickness, and hemorrhage [83]. In spite of the high therapeutic potential of Epo, further studies need to define the optimal dose and timing of Epo administration to treat both preterm-associated pathologies, i.e., severe lung injury and long-term neurodevelopmental disorders. This is supported by the randomized Preterm Erythropoietin Neuroprotection Trial (PENUT) trial in preterm infants, revealing only a trend towards a lower rate of death and improved neurodevelopmental outcome at two years of corrected age. However, considering long-lasting ongoing neurodevelopmental processes in childhood, long-term follow-up analyses of neurodevelopment are needed [153].

## 7. Stem Cell-Based Therapeutic Approaches in Neonatal Lung and Brain Injury

For more than a decade mesenchymal stromal/stem cells (MSC) have shown promising therapeutic properties in regenerative medicine [154, 155]. MSC are multipotent cells with immunomodulatory properties [156, 157] and are capable of self-renewal (to proliferate without losing their differentiation potential) [155]. They can be easily isolated from bone marrow (BM), adipose, and placental tissue. For treating neonatal diseases such as BPD and EoP, MSC isolated from the umbilical cord (Wharton jelly) or umbilical cord blood (UCB) gained increasing interest for therapeutic purposes, because their use is ethically acceptable, painless for infant and mother, and the UCB is otherwise discarded tissue [23, 24]. A roadmap for translating MSC therapy into clinical practice has been recently summarized and discussed [158]. Until now, 80% of ongoing clinical trials investigating MSC administration in neonatal brain injury used UCBC (stem and progenitor cell) and UCB-derived MSC, even though data on outcome have not been published so far [159].

While only few experimental studies exist analyzing the impact of MSC-administration in hyperoxia-induced brain injury [45], several reports demonstrated beneficial effects of MSC in models of hypoxic-ischemic (HI) brain injury. For example, MSC-treatment restored myelination and attenuated neurodegeneration in grey matter lesions associated with an increased proliferation of neuronal and oligodendrocyte precursor cells and an improved oligodendrocyte maturation [160, 161]. Furthermore, enhanced white and grey matter structure was associated with improved long-term cognitive and sensorimotor functions, reduced microglial and astroglial activation after MSC-treatment in HI-induced brain injury in neonatal mice [162, 163]. Similar protective effects of MSC-treatment were demonstrated in preclinical BPD models, revealing that hyperoxia-induced alveolar simplification and vessel degeneration can be improved by an early MSC administration [66]. In addition to improved lung and brain structure, MSC therapy reduces injury-associated inflammatory responses in both tissues, e.g., decreased proinflammatory cytokine expression [45, 161, 163, 164]. So far, only one experimental study investigated the effects of MSC therapy on hyperoxia-induced tissue injury in both organs in the same experimental setting [45]. Although MSC could not be detected in the brain, intratracheal administrated MSC simultaneously attenuated impaired lung development and restored myelination, as demonstrated by enhanced alveolar growth and increased amounts of mature (MBP^+^) oligodendrocytes, respectively [45]. A recently suggested mechanism that might be involved in these remote protective processes is MSC-derived extracellular vesicles (MSC-EV). MSC-EV have been shown to ameliorate experimental BPD and restore lung function through macrophage immunomodulation [165]. Similarly, in models of neonatal brain injury, MSC-EV revealed neuroprotection involving immunomodulatory and anti-inflammatory mechanisms [166–168].

Promising experimental findings led to the initiation of several clinical trials to evaluate stem-cell based regenerative therapies in preterm-associated morbidities. To test, whether intratracheally administrated MSC are safe and feasible for preventing BPD, 9 preterm infants at high risk for developing BPD received a single dose of 1∗10^7^ or 2∗10^7^ allogeneic human UCB-derived MSC/kg in a phase I dose escalation trial [169]. Compared to a historical control group, a slight reduction of BPD severity in MSC-treated infants was noticed up to two years corrected age without serious adverse events including tumorigenicity [169, 170]. None of the treated infants developed cerebral palsy and neurodevelopmental outcome was comparable to historical controls. Therefore, the authors concluded that attenuation of BPD severity by an early MSC treatment may ameliorate neurodevelopmental morbidities such as cerebral palsy frequently associated with BPD [170]. A long-term follow-up study until the age of 5 years and a phase II clinical trial are currently conducted (NCT02023788). Recently, results of a phase II trial were published from the same group, showing safety of the treatment but no significant beneficial effect [171]. For neonatal brain injury, Cotten et al. showed that repetitive intravenous infusions of autologous cord blood cells were feasible and safe in a small group of hypothermia-treated infants with hypoxic-ischemic encephalopathy [172]. Another study demonstrated the safety and feasibility of an intraventricular transplantation of a single dose of 1∗10^7^ or 2∗10^7^ UCB-MSC/kg in preterm infants with severe IVH [173]. Currently, further clinical trials are ongoing for perinatal brain injury to prove clinical feasibility of MSC therapy (NCT03635450, NCT02434965, NCT02881970, NCT02612155, NCT02673788, and NCT02890953). Nevertheless, the majority of trials focuses on injury in term-born infants while only few studies address preterm-birth-related neurological disorders.

In addition to safety and feasibility, major challenges related to regenerative stem cell-based therapies include the definition of the optimal source, dose, route, and time point of administration. MSC can easily be isolated from various tissues with different properties [174]. In addition to bone marrow-derived stem cells (BMSC), MSC derived from the UC and the UCB are increasingly discussed for autologous treatment of neonatal brain and lung injury [159, 175]. However, MSC's therapeutic capacities may be negatively influenced by preterm-birth related complications. Recent studies in immunological disorders revealed that MSC's function depends on the (micro)environment. For example, in Graft-versus-Host disease models, it was shown that BM-MSC are less protective in the secondary disease phase when resolution of inflammation is induced, suggesting that MSC need to get activated by their environment to promote protection [176]. This is also supported by recent work in neonatal brain injury, where hypothermia-induced changes in the brain microenvironment in HI-injured animals limited MSC's protective capacity [161]. The complexity of MSC function depending on the condition they are exposed to is also reflected by previous work about limited functionality of endogenous lung MSC in animals exposed to hyperoxia [177]. These data highlight plasticity of MSC function in a context-dependent manner, which needs to be considered in autologous transplantation approaches of UC- and UCB-derived MSC in case of prematurely born infants. Due to the discussed potential limitations of autologous MSC therapy in critically affected preterm born infants, time-consuming *ad hoc* MSC-isolation and expansion, allogeneic MSC from UC and UCB of healthy (pre)term-born infants may be the preferred therapeutic approach [23, 174], which needs to be proven in future studies.

Besides optimal sources, the cell number, delivery routes, and therapeutic windows for efficient treatment effects need to be defined. Several experimental studies tested a single dose with varying cell numbers between 5∗10^3^ and 1∗10^6^ cells/animal [45, 66, 160, 161, 164]. Currently, no guidelines are available to extrapolate the dose of cells used in experimental studies to clinical trials [178]. In the clinical setting, MSC doses between 1∗10^7^ and 2∗10^7^ cells/kg were administered [169]. Chang and colleagues showed that the respiratory severity score decreased three days after treatment, but subsequently increased at day 7, indicating that a second MSC administration is necessary [169]. Furthermore, the amount of MSC may depend on the route of administration. Several experimental BPD studies prefer local intratracheal (i.t.) MSC application [45, 66, 164], which can be administrated similar to surfactant therapy. However, with regard to brain injury intraperitoneal (i.p), intranasal (i.n.) or intraventricular administration was mainly applied in experimental studies [161–163]. Liu et al. revealed that i.p. administration of MSC has a dose-depended effect (highest and most effective dose: 1∗10^6^ MSC/animal) in hyperoxia-induced lung injury whereas even the highest dose of i.n. administrated MSC showed no protective effects in the lung [128]. A major challenge is the definition of an optimal therapeutic window for MSC therapy. Several preclinical studies analyzed the effect of an early and late administration for prevention and regenerative therapy, respectively [66, 162, 163]. MSC administration was most effective when given at early time points, i.e., 3 days after neonatal HI-induced brain injury and at P4 in models of BPD. The therapeutic potential declined, when administered at later time points (i.e., 7 or 10 days after HI-induced brain injury and at P14 in BPD models) [66, 162, 163]. Taken together, the optimal treatment regime needs to be defined, ideally in combined analyses of both organs in the same experimental setting of hyperoxia.

## 8. Conclusion and Perspectives

Improved neonatal care significantly increased survival of premature born infants in the last decades. While severe life-threatening diseases like IVH and CP declined, the incidence of BPD and EoP remained high. A major contributing factor linking both pathologies is the exposure of an extra-uterine fetus to higher oxygen concentrations, leading to impaired lung and brain development. In support of this, a large number of experimental studies revealed pronounced similarities in tissue-damaging pathways, e.g., oxidative stress and inflammation. To date, only few studies focused on analyses of both organs in the same experimental setting. Even though these simultaneous analyses would provide further important insight into pathology affecting the lung and the brain in preterm-born children, cause and consequence can hardly be distinguished. Further research to disentangle the complex interrelationship between both organs is needed to answer the question whether the lung-brain-axis exists in preterm-related comorbidities. One approach will be the inclusion of cell-specific *in vitro* analyses with brain and lung-derived cells, which can reveal the direct impact of hyperoxia on these cells. However, to investigate the connection between these two organ systems in a complex organism, aligned *in vivo* models are needed. These improved models may allow the development of novel therapeutic approaches. Even though a large body of preclinical studies suggested different therapeutic options, a common therapy to simultaneously treat both organ injuries is still missing. Pharmaceutical (e.g., caffeine) but also stem cell-based multimodal therapies seem promising to prevent both, lung and brain injury. Therefore, intense clinical and preclinical research, also including large animal models, is needed to define the optimal treatment design (i.e., dose, timing, and administration route) and to develop rational therapy designs targeting both preterm birth-related diseases, i.e., BPD and EoP.

## Figures and Tables

**Table 1:** Experimental models of hyperoxia-induced brain and lung injury.

	O_2_ concentration (%)	HO onset	HO duration	Species	Reference
Brain	40-80	P7	2 h–3 d	Wistar rats/ synRAS mice and wt mice	[43]
	80	Birth	6 d	Sprague-Dawley rats	[44]
	80	P1	7 d	Sprague-Dawley rats	[69]
	80	P3	48 h	Wistar rats	[50]
	80	P3, P6, P10	24 h	Wistar rats	[75]
	80	P6	2 h–48 h	Wistar rats	[53, 73, 101]
	80	P6	2 h–48 h	C57BL/6^IRAK-4[-/-]^ and C57BL/6 mice	[53]
	80	P6	12 h, 48 h	Wistar rats	[72]
	80	P6	24 h	Wistar rats	[49, 54, 74, 108, 134–136, 152, 179]
	80	P6	24 h	SynRas mice and C57BL/6	[57]
	80	P6	6 h–48 h	Wistar rats	[52]
	80	P6	24 h, 48 h	Wistar rats	[51]
	80	P6	48 h	C57B/6J mice	[55, 56]
	≥80	P7	24 h	Wistar rats	[98]
	85	P2	12 d	C57BL/6 mice	[70]
	95	P0	7 d	C57BL/6 mice and C57BL/6^(hEC-SOD)^ mice	[71]
	>95	P5	7 d	Sprague-Dawley rats	[42]
	100	P0	4 d	C57BL/6J mice and Sftpc^(EC−SOD)^ mice	[68]

Lung	60	P1	14 d	Sprague-Dawley rats	[180]
	65	P3	4 weeks	C57BL/6J mice	[91]
	70	P0	14 d	C57BL/6J mice (male)	[62]
	75	P1	7 d	FVB mice	[165]
	80	P0	3 d, 5 d	Wistar rats	[59, 102]
	80	P1	10 d	C57BL/6J mice	[99]
	80	P6	6 h-48 h	Wistar rats	[100]
	40-80	P0	3 d–28 d	C57BL/6 mice and B6.129S2-IL6^(tmlKopf/j)^	[60]
	40-85	P1, P4	24 h–14 d	C57BL/6 mice	[76]
	85	P0	14 d	C57BL/6J mice	[61]
	85	P1	14 d	C57BL/6J, BALB/cJ, FVB/NJ, C3H/HeJ, DBA/2J, 129S2/SvPasOrlRj mice	[77]
	85	P0	10 d	C57BL/6 mice	[129, 181]
	85	P0	28 d	C57BL/6J, C57BL/6N mice	[78]
	85	P1	14 d	C57BL/6 mice	[182]
	85	P1	28 d	C57BL/6 mice	[119]
	85	P3	12 d	Nlrp3^−/−^ and WT mice	[106]
	90	Birth	7 d	SCID-mice	[128]
	90	Birth	14 d	Sprague-Dawley rats	[169]
	90	P1	10 d	Sprague-Dawley rats	[183]
	≥90	P3	10 d	Wistar rats	[148]
	95	Birth	14 d	Sprague-Dawley rats	[66]
	95	Birth	14 d	Sprague-Dawley rats	[65]
	95	Birth	14 d	rats	[64]
	95	P0	7 d	C57BL/6 and SPC hEC-SOD TG mice	[58]
	95	P1	6 d	Sprague-Dawley rats	[146]
	>97	<P2	3 d–15 d	Sprague-Dawley rats	[92]
	96-100	P0	8 d	Sprague-Dawley rats (male)	[63]
	100	P0	4 d	Sftpc-EGFP mice, Rat Scgb1a1-rtTA and (otet)7CMV-cre bitransgenic mice x mT/mG mice	[67]
	100	P2	9-10 d	Wistar rats	[122]

Brain and lung	80	Birth	7 d	Sprague-Dawley rats	[48]
	85	P1	10 d	C57BL/6J mice	[47]
	85	P1	14 d	Sprague-Dawley rats	[46]
	90	P0	14 d	Sprague-Dawley rats	[45]

## Data Availability

No data were used to support this study.
